# Essential Coaching for Every Mother Tanzania (ECEM-TZ): Protocol for a Type 1 Hybrid Effectiveness-Implementation Randomized Controlled Trial

**DOI:** 10.2196/63454

**Published:** 2024-12-05

**Authors:** Justine Dol, Lilian Teddy Mselle, Marsha Campbell-Yeo, Columba Mbekenga, Thecla Kohi, Douglas McMillan, Cindy-Lee Dennis, Gail Tomblin Murphy, Megan Aston

**Affiliations:** 1 Faculty of Health Dalhousie University Halifax Canada; 2 IWK Health Halifax, NS Canada; 3 School of Nursing Muhimbili University of Health and Allied Sciences Dar es Salaam United Republic of Tanzania; 4 School of Nursing Dalhousie University Halifax, NS Canada; 5 School of Nursing Kairuki University Dar es Salaam United Republic of Tanzania; 6 Faculty of Medicine Dalhousie University Halifax, NS Canada; 7 Lawrence S Bloomberg Faculty of Nursing University of Toronto Toronto, ON Canada; 8 Department of Psychiatry University of Toronto Toronto, ON Canada; 9 Lunenfeld-Tannenbaum Research Institute Sinai Health Toronto, ON Canada; 10 Nova Scotia Health Halifax, NS Canada

**Keywords:** mobile health, maternal health, randomized controlled trial, parenting self-efficacy, self-efficacy, maternal, RCT, mother, text message, coaching, postnatal, newborn, child, low-income country, middle-income country, Africa, newborn care education, nurse midwife, Tanzania

## Abstract

**Background:**

Despite global goals to improve maternal, newborn, and child health outcomes, mortality and morbidity continue to be a concern, particularly during the postnatal period in low- and middle-income countries. While mothers have the responsibility of providing ongoing care for newborns at home, they often receive insufficient newborn care education in Tanzania. Mobile health via text messaging is an ever-growing approach that may address this gap and provide timely education.

**Objective:**

We aim to evaluate a text message intervention called Essential Coaching for Every Mother Tanzania (ECEM-TZ) to improve maternal access to essential newborn care education during the immediate 6-week postnatal period.

**Methods:**

ECEM-TZ consists of standardized text messages from birth to 6 weeks post partum that provide evidence-based information on caring for their newborn and recognizing danger signs. Messages were developed and then reviewed by Tanzanian mothers and nurse midwives before implementation. A hybrid type 1 randomized controlled trial will compare ECEM-TZ to standard care among mothers (n=124) recruited from 2 hospitals in Dar es Salaam. The effectiveness outcomes include newborn care knowledge, maternal self-efficacy, breastfeeding self-efficacy, maternal mental health, attendance at the 6-week postnatal checkup, and newborn morbidity and mortality. The implementation outcomes include the reach and quality of implementation of the ECEM-TZ intervention.

**Results:**

Recruitment for this study occurred between June 13, 2024, and July 22, 2024. A total of 143 participants were recruited, 71 in the control and 72 in the intervention. The 6-week follow-up data collection began on July 30, 2024, and was completed on September 21, 2024.

**Conclusions:**

This study will generate evidence about the effectiveness of implementing text messaging during the early postnatal period and the feasibility of doing so in 2 hospitals in Dar es Salaam. The intervention has been designed in collaboration with mothers and nurse midwives in Tanzania.

**Trial Registration:**

ClinicalTrials.gov NCT05362305; https://clinicaltrials.gov/study/NCT05362305

**International Registered Report Identifier (IRRID):**

DERR1-10.2196/63454

## Introduction

### Background

The postnatal period during the first 6 weeks of life is a critical time point for newborns and mothers in Tanzania, which has a neonatal mortality ratio of 20 per 1000 live births [1] and a maternal mortality ratio of 104 per 100,000 live births [2]. Neonatal and maternal mortality is highest in the first week post partum [3,4]. Despite the high number of deaths that occur postnatally, most mothers and their newborns receive insufficient care during this period, especially in low- and middle-income countries (LMICs) [5]. There is a significant need to develop and evaluate innovative approaches to reach mothers during this period to ensure they are knowledgeable and confident in caring for their newborns at home.

While most Tanzanian women (90%) have at least 1 antenatal care contact, only half (51%) of the women and 54% of the infants receive postnatal care within 2 days after giving birth [2]. Further, health facilities are often unable to provide individualized education based on maternal needs due to staff shortages and resource constraints [6-8]. Across Tanzania, mothers receive little education about how to care for their newborns before hospital discharge due to significant health system challenges [6,8,9].

One innovative strategy that can be used to provide postnatal education directly to parents is mobile health (mHealth) [10]. The goal of mHealth interventions delivered postnatally is not to replace the need for in-person follow-up but to complement existing care and fill knowledge gaps by providing timely, standardized, relevant, and evidence-based information on newborns’ care at home. The use of mobile phones to reach mothers is supported by the World Health Organization recommendations, which state that traditional postnatal contact can be enhanced through mobile phone–based contact between mothers and the health system [11].

Due to the severe shortage of health care providers and the low rate of in-person postnatal contacts in Tanzania [12], Essential Coaching for Every Mother Tanzania (ECEM-TZ) is poised to bridge this gap without adding a significant burden to the health system. The goal of the ECEM-TZ intervention is to improve maternal access to knowledge during the immediate 6-week postnatal period, and to increase maternal self-efficacy and improve mental health outcomes.

### Effectiveness Objectives

The objective is to compare mothers who receive ECEM-TZ to mothers who receive standard care on newborn care knowledge, maternal parenting self-efficacy, breastfeeding self-efficacy, postpartum depression symptoms, anxiety symptoms, postnatal care attendance, newborn morbidity, and newborn mortality at 6 weeks post partum.

### Effectiveness Hypotheses

Mothers who receive ECEM-TZ will have higher levels of newborn care knowledge, maternal parenting self-efficacy, breastfeeding self-efficacy, and postnatal care attendance and lower rates of postpartum depression symptoms, anxiety symptoms, and newborn morbidity and mortality at 6 weeks post partum in comparison to those who received standard postpartum care.

### Implementation Objectives

The objective is to explore the implementation extent and quality of ECEM-TZ*.* Using process evaluation guidelines [13,14], the specific questions for ECEM-TZ are as follows:

1. *Recruitment effectiveness*: was this study able to recruit the desired number of participants in the time frame? Is there a difference in recruitment based on hospital level? What is the recruitment rate (ie, approached vs randomized)?
2. *Implementation extent*: what was delivered, including the quality (fidelity), dose delivered (completeness), dose received (exposure), satisfaction, reach (participation rate), and recruitment?
3. *Implementation quality*: what were the barriers and facilitators in implementing behavior change based on ECEM-TZ content? What is the level of satisfaction of ECEM-TZ?

## Methods

### Study Design

This is a 2-arm, parallel-group, hybrid type 1 randomized controlled trial (RCT). Participants will be randomized to receive a 6-week postpartum text message program called ECEM-TZ or standard care (no messages) with a 1:1 allocation*.*

A hybrid type 1 RCT study focuses on evaluating clinical outcomes while also seeking to understand the context for future implementation through a process evaluation [15,16]. For this protocol, a hybrid type 1 RCT study was selected as ECEM-TZ is a new digital health solution and thus, exploring whether the intervention improves clinical outcomes is the key focus. However, it is also important to explore the implementation factors related to extent and quality as well as recruitment effectiveness to ensure that the intervention effectiveness outcomes are not put at risk or misinterpreted if the digital health solution is not able to be implemented as intended.

### Intervention

#### ECEM-TZ Development (Pre-RCT Phase)

To create a standardized, evidence-based approach to the content, the initial messages were developed using the World Health Organization guidelines for postnatal care [11]. There were 54 unique messages initially developed: 6 related to the research study, 6 on postnatal care, 7 on feeding, 3 on handwashing, 3 on cord care, and 4 on thermal care. For messages related to danger signs, 5 each were on fever and breathing with 3 each on feeding, no movement, jaundice, cord infection, and convulsions. ECEM-TZ messages were designed to be tailored to each participant in that all mothers will receive the same content but may include the name of the mother or child. All text messages were developed in English and then translated into Kiswahili using standardized forward-backward translation processes [17] before testing.

All messages mapped onto the social cognitive theory of behavior change [18,19] and included four types: (1) educational, (2) reminders, (3) social support, and (4) interactivity (two-way), all to reinforce positive behaviors associated with newborn care. Educational messages provided one-way information about essential newborn care to encourage action, such as exclusive breastfeeding and hygiene cord care. Reminder messages consisted of one-way messages about upcoming postnatal contacts and returning to a clinic if they recognize a danger sign. Social support messages included messages from other fictional mothers encouraging them to provide evidence-based care. Finally, interactivity (two-way) messages included options for mothers to respond to inquiries, such as checking into whether they visited a postnatal clinic within the past seven days.

To ensure that the content of ECEM-TZ was appropriate and acceptable, the messages underwent two rounds of iterative testing with Tanzanian mothers and nurse midwives. Mothers were eligible for inclusion if they met the following criteria: (1) had recently given birth within the hospital; (2) were aged older than 18 years; and (3) spoke and read English or Kiswahili. Mothers were excluded if (1) their newborn died or was expected to die before leaving the hospital or (2) they were experiencing major postnatal complications expected to impact their ability to provide informed consent (eg, major postpartum hemorrhage or seizures). Nurse midwives were eligible to participate in this study if they (1) worked in a hospital on a labor or postnatal ward for at least three months to ensure that they had experience providing care to this population, (2) were currently involved in providing maternal and newborn care either on the labor ward or postnatal unit, and (3) could speak and read English or Kiswahili. Nurse midwives were excluded if they were not on duty during the data collection period.

On recruitment and before starting the interview, oral and written consent was obtained for this study’s purpose, as well as consent for the use of a voice recorder by all participants. Interviews were conducted by a research assistant at the hospital in a comfortable location as suggested by the participant. Participants were shown messages based on the topic area and asked: “What do you think this phrase is saying? Does anything come to mind when you read this? Are there any words that you did not understand? Was anything in this unacceptable or offensive? Are there any words you would suggest that could be used instead? If so, which one conforms best to your usual language?” Mothers were also asked to review and complete the Karitane Parent Confidence Scale [20], which was translated into Kiswahili. Interviews were transcribed verbatim and translated into English by a Tanzanian research assistant to facilitate analysis. A summary of concerns for both the Karitane Parent Confidence Scale and ECEM-TZ was created during each round of testing. Changes were made to the text messages based on participant feedback and any revisions were retranslated in Kiswahili. Final messages were translated back into English for backward translation to ensure the message remained intact and evidence-based [17].

In total, 10 mothers and 7 nurse midwives participated in the validation. In round 1, three mothers and 3 nurse midwives provided feedback, and in round 2, seven mothers and 4 nurse midwives provided feedback. Mothers were on average aged 27.4 (SD 5.1) years and had 1 (SD 1.4, range 0-4) other child. Nurse midwives were on average aged 37.4 (SD 8.9) years and had 10.8 (SD 6.9, range 1-19) years of clinical experience.

#### Final Intervention

ECEM-TZ is a one-way text message program sent from the third day after birth until 6 weeks postnatally. Messages are sent once per day in the morning. After 2 rounds of testing, the ECEM-TZ was finalized with a total of 44 messages that covered the following topics: 4 related to the research study, 7 on feeding, 6 on postnatal care follow-up, 4 on normal development, 3 each on thermal care and maternal care, and 2 each on cord care and handwashing. For messages related to danger signs, 3 each were on fever, no movement, or convulsions; 2 each were on poor feeding, jaundice, and breathing; and 1 was on cord infection. Given participant feedback during the pretesting, social support and two-way messages were removed due to reported poor participant acceptance and perceived clarity. Thus, only educational and reminder messages were included. No changes in care or education will be implemented, with mothers in the intervention group receiving standard in-hospital education as provided to all women delivering at that hospital before discharge from the postpartum unit. Mothers will be able to message “acha” (stop in Kiswahili) to withdraw from this study at any time and will stop receiving messages.

### Control Group

No changes in care or education will be implemented, with mothers in the control group receiving standard in-person education as provided to all women delivering at that hospital before discharge from the postpartum unit. Mothers will not receive any text messages from the program.

### Procedure (RCT)

Mothers will be recruited by a research assistant after giving birth at a health care facility and before discharge. Mothers will be approached and informed about this study. If interested, mothers will be screened against eligibility criteria. Eligible and consenting mothers will complete a baseline questionnaire (T1) and are then randomized into a study group. All participants will be followed up at six weeks postnatally (T2) via telephone.

### Randomization, Allocation, and Blinding

Using a 1:1 allocation, participants will be randomized into either the intervention or control group using a computer-generated stratified block randomization with blocks varying between 4 to 8 via REDCap (Research Electronic Data Capture; Vanderbilt University). Stratification based on the site of recruitment will be implemented. Due to the nature of ECEM-TZ, blinding will not be possible for mothers (ie, mothers will know if they receive text messages or not). To minimize bias at the participant level, all mothers will receive the same in-hospital care. Due to the nature of the intervention, mothers in the control group will not be exposed to the intervention text messages. Hospital staff (nurse midwives, physicians, and other health care providers) will be blind to participant group allocation to ensure both groups receive the standard discharge education. The researchers will be aware of participant allocation but will not be involved in data collection from participants or the randomization process. The research assistants will be blinded to allocation at baseline (T1) but not at T2.

### Setting

This study is being conducted in Dar es Salaam, Tanzania, which is in East Africa. Participants will be recruited from the Mwananyamala Regional Hospital and Sinza Palestina Hospital. The Mwananyamala Regional Hospital is a large government-run hospital that has 70 to 90 deliveries per day. The Sinza Palestina Hospital is a governmental, public hospital that has up to 50 deliveries per day.

### Inclusion Criteria

Mothers are eligible if they meet the following criteria: (1) have given birth within the past 48 hours, (2) have daily access to a mobile phone with text messaging capabilities, (3) are aged older than 18 years, and (4) speak and read English or Swahili. Participants will be excluded if (1) newborns die or are expected to die before leaving the hospital; (2) they have no access to a mobile phone, either personal or shared; (3) they are unwilling to receive SMS messages; (4) decline to participate; (5) are experiencing major postnatal complications expected to impact learning or ability to consent while in the hospital (eg, postpartum hemorrhage or seizures); or (6) participated in ECEM-TZ development phase.

### Ethical Considerations

Ethics approval has been received from Dalhousie University (REB#2018-4538), Muhimbili University of Health and Allied Science (REB#MUHAS-REC-7-2019-010), and National Medical Institute of Research (REB#NIMR/HQ/R.8c/Vol.I/2457). All participants will be provided with written and verbal information and will complete a written consent form before enrollment in the study. To maintain confidentiality, all study data will be identified using a study number unique to each participant on all questionnaires and surveys. All data will be entered into REDCap by a research assistant in Tanzania with the first author having access to the data. REDCap is a secure web application for building and managing online surveys and databases [21]. All participants will receive the equivalent of honorarium (TZS 10,000; approximately US $4) in phone vouchers to their airtime provider of choice.

### Outcome Measures

Baseline data will be collected in person in the hospital while follow-up data at 6 weeks post partum will be collected by telephone. Research assistants will attempt to contact participants until 10 weeks post partum to obtain complete follow-up data. Table 1 shows the outcome assessment time points.

**Table 1:** Outcome assessment time points.

Outcome	Baseline (T1^a^)	6-Week follow-up (T2)
Eligibility screen	✓	
Informed consent	✓	
Randomization	✓	
Demographics	✓	
Newborn care knowledge	✓	✓
Parenting self-efficacy	✓	✓
Breastfeeding self-efficacy	✓	✓
Postpartum anxiety	✓	✓
Postpartum depression	✓	✓
Neonatal morbidity and mortality		✓
Postnatal clinic attendance		✓
Implementation quality—evaluation survey		✓
Implementation extent	✓	✓

^a^T: time point.

### Effectiveness Outcomes

To measure the effectiveness of ECEM-TZ, the following will be assessed:

1. Newborn care knowledge: Knowledge will be assessed using a modified questionnaire developed by McConnell and colleagues [22] assessing newborn care knowledge. The questions determine whether mothers can identify danger signs, handwashing practices, cord care, newborn thermal care, and breastfeeding. A total sum of the number of items mothers can identify will be created. Additionally, binary variables will be created to determine whether mothers can identify three or more newborn danger signs, two or more best practice hand washing, two or more cord care practices, three or more thermal care practices, and two or more breastfeeding practices. Based on these variables, a summative score will be created ranging from 0 to 5, with a higher score representing increased newborn care knowledge.
2. Maternal self-efficacy: Parenting self-efficacy will be measured using the Karitane Parenting Confidence Scale [20]. This 15-item scale was developed to assess the perceived self-efficacy of mothers of newborns from birth to aged twelve months and has acceptable internal consistency (Cronbach α=0.81) and test-retest reliability (*r*=0.88) [20]. A cutoff score of 39 or less (out of a possible 45) was determined to be a clinically low perceived parenting self-efficacy [20]. The Kiswahili version was piloted as part of the development phase.
3. Breastfeeding self-efficacy: Breastfeeding self-efficacy will be measured using the Breastfeeding Self-Efficacy Scale—Short Form [23]. This 14-item tool was developed originally in Canada and has been translated into Kiswahili and determined to be valid and reliable [24]. Higher scores indicate higher breastfeeding self-efficacy.
4. Postpartum depression: Depression will be measured using the Edinburgh Postnatal Depression Scale (EPDS) [25]. The EPDS is a self-report screening scale with 10 items that can indicate if a respondent has symptoms related to perinatal depression [25,26]. The EPDS is valid for assessing depressive symptoms across the perinatal period with a score >12 considered as high symptoms of depression. The EPDS has been used in a variety of African countries to measure perinatal depression [27-29]. The EPDS has already been translated into Kiswahili and has been modified for contextual differences [30].
5. Postpartum anxiety: Anxiety will be measured using the Generalized Anxiety Disorder (GAD)-7 [31], which is a 7-item scale used to assess GAD. The scale ranges between 0 and 21, with scores of 10 or greater indicative of generalized anxiety at a moderate or severe level. The GAD-7 has already been translated into Kiswahili [32] and has been widely used to measure perinatal anxiety in African countries, including in Tanzania [33,34].
6. Newborn morbidity and mortality: The 6-week follow-up survey will collect information on morbidity (eg, diarrhea, jaundice, or infection), need for readmission due to illness, and mortality.
7. Postnatal clinic attendance at six weeks: The 6-week follow-up survey will collect information on the number and date of postnatal contacts.

### Implementation Outcomes

To measure the implementation of ECEM-TZ, 3 measurement tools will be used.

1. Data related to recruitment will be collected, such as the number of women approached at each site and the number of women randomized.
2. Output data available through the Twilio and TextIt platforms will be collected per participant, including the number of messages sent, messages delivered, error messages, opt-out rates, and response rates.
3. At the final follow-up at 6 weeks post partum, mothers in the intervention group will evaluate the program and be asked about the number of messages they recall receiving, user experience, perspectives on the frequency and timing of messages, and what they liked and did not like about ECEM-TZ.

### Sample Size

The sample size was calculated based on the outcome of newborn care knowledge. While previous studies on postnatal education knowledge are significantly varied and no studies to date have reported on maternal postnatal education knowledge in Tanzania, we considered a binary summative score of 100% as the goal at 6 weeks post partum. Using a power of 80%, α of .05 (2-tailed), and assuming 90% of the intervention group and 70% of the control group will score 100% at 6 weeks, a total sample size of 124 is required. This is in line with our implementation sample size approach: recruiting across two hospitals to determine the feasibility of recruitment at these sites. We will aim for approximately similar numbers of participants at each site, recruiting for six weeks. Assuming two research assistants are recruiting five days per week at each hospital and each research assistant can recruit a minimum of 2 participants per day, this would be approximately 20 participants per week. Over six weeks, this would be 120 participants we anticipate recruiting. If recruitment is higher than anticipated, it will be limited to a maximum of 200 across both sites.

### Statistical Analysis

Data will be analyzed on an intention-to-treat (ITT) analysis basis as well as per protocol analysis (excluding women who requested to stop the program or were lost to follow-up). Data will be analyzed using SPSS (version 29, IBM SPSS Statistics, IBM Corporation). Demographic characteristics will be expressed in means, SDs, and percentages, as applicable, based on group allocation. Any significant differences in baseline characteristics, examined through a chi-square analysis or Mann-Whitney test, will be adjusted for in the analyses. A *P* value of .05 will be considered significant for all outcomes. For all analyses, unadjusted and adjusted models will be reported.

For the effectiveness outcomes, total and summative scores will be reported using means, SDs, and percentages, as appropriate. Analysis of covariance will be used to measure whether scores differ between the two groups, adjusted for the pretest scores and any significant baseline characteristics. For newborn care knowledge, newborn morbidity, newborn mortality, and attendance at postnatal care six-week contact, binary variables will be created, and data will be analyzed using logistic regression.

For the implementation outcomes, descriptive statistics will be used to describe mothers’ experience with ECEM-TZ and outcomes will be reported using means, SDs, and percentages, as applicable. Open-ended questions will be analyzed using thematic analysis [35]. Responses will be transcribed verbatim and translated into English for analysis.

## Results

**Figure 1:**
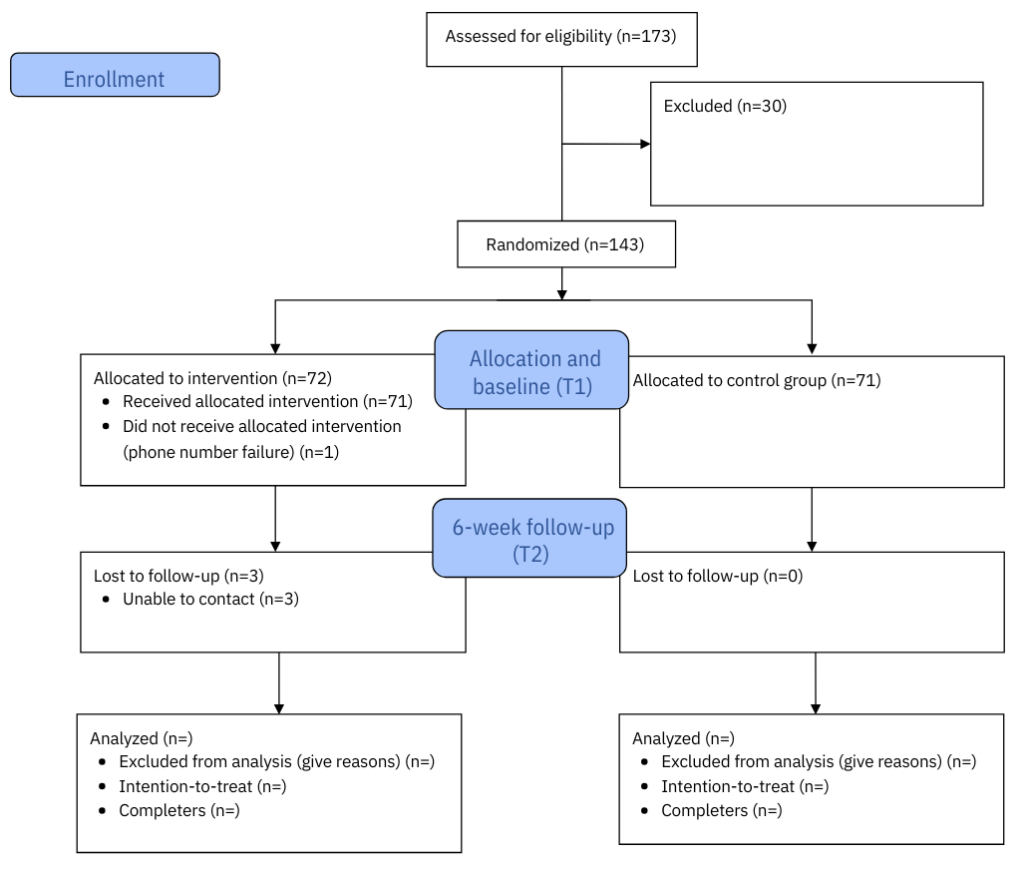
CONSORT flow diagram. CONSORT: Consolidated Standards of Reporting Trials. As data analysis has not yet been completed, the final numbers for analysis has not yet been confirmed. The completed CONSORT will be available once the results are published.

Recruitment for this study occurred between June 13, 2024, and July 22, 2024. A total of 143 participants were recruited, 71 in the control and 72 in the intervention. The 6-week follow-up data collection began on July 30, 2024, and was completed on September 21, 2024. Figure 1 outlines the CONSORT (Consolidated Standards of Reporting Trials) flow diagram.

## Discussion

### Anticipated Findings or Significance

We anticipate the mothers who receive ECEM-TZ will report a significant increase in newborn care knowledge compared to those who only receive standard care. We also anticipate mothers in the intervention group will have higher self-efficacy, be more likely to attend their 6-week follow-up postnatal clinic visit, and have lower depression and anxiety symptoms and fewer newborn mortality and morbidity outcomes at 6 weeks post partum. These results, if present, suggest that providing mothers additional newborn care knowledge to complement in-hospital postnatal education through text messages during the first 6 weeks after birth can improve maternal and newborn outcomes for mothers in Tanzania.

### Comparison to Prior Work

Providing text message support to mothers in Tanzania is not new. For example, in 2012, *Wazazi Nipendeni* (“Love me, parents” in Kiswahili) was an initiative in Tanzania that sought to improve maternal health outcomes by sharing health-promoting messages through radio, television shows, print advertisements, and billboards [36]. It was developed by the Johns Hopkins Bloomberg School of Public Health Center for Communication Programs and led by the Ministry of Health and Social Welfare [37]. As a subcomponent of *Wazazi Nipendeni,* the text message component of Healthy Pregnancy, Healthy Baby had the objective of sharing antenatal and nutrition information to mothers and their partners, friends, and relatives (as requested) via text message between pregnancy and 16 weeks post partum [38,39]. Mothers were sent three to four text messages per week on topics including preventing mother-to-child transmission of HIV/AIDS, antenatal and postnatal care, family planning, malaria prevention, nutrition, and danger signs, all of which were developed and approved by the Government of Tanzania’s Ministry of Health and Social Welfare, targeted toward the gestational age of the newborn [38]. However, during the evaluation, only 8.8% of women recalled exposure through text messages [36].

*Wired Mothers* is another example of an mHealth intervention that was developed and evaluated in Zanzibar, a semiautonomous island off the coast of Tanzania, through collaboration with University of Copenhagen, Zanzibar’s Ministry of Health and Social Welfare, and the Danida Health Sector Programme Support [40]. Targeting pregnant mothers (recruited at their first antenatal care contact) to 42 days post partum, a cluster RCT was conducted with 2550 women [41]. The *Wired Mothers* intervention had two components: unidirectional, automatic text messages to mothers with registered numbers and a voucher system to allow for two-way communication between mothers and their health care providers [41]. The text messages contained health information related to pregnancy, skilled delivery, and postnatal care as well as reminders to attend antenatal care contacts according to the gestational age of the newborn [41]. The *Wired Mothers* intervention significantly improved the likelihood of women having more antenatal contacts, delivering with a skilled birth attendant for urban women, and reducing perinatal mortality risk [41,42].

However, these text message interventions were developed and evaluated over a decade ago (2012-2014), and neither focus on maternal newborn care knowledge, self-efficacy, or other mental health outcomes such as depression and anxiety. Thus, there is a need to evaluate ECEM-TZ as a potential to improve maternal outcomes in these dimensions. In a recent systematic review of mHealth interventions targeting the perinatal period in LMICs, it was found that only a few studies reported on self-efficacy and knowledge outcomes [10]. Furthermore, a recent study in Tanzania found that in the immediate postpartum period, 25% of women experienced depression symptoms and 37% experienced anxiety symptoms [43]. This indicates that exploring the potential of mHealth interventions that target psychosocial and mental health outcomes in LMICs is needed to improve maternal psychosocial and mental health outcomes.

### Strengths and Limitations

This protocol has several strengths that should be acknowledged. First, mothers and nurse midwives in Tanzania were involved in providing feedback on the content of the messages before implementation. The messages were also designed using the World Health Organization guidelines for postnatal care [11] and mapped onto the social cognitive theory of behavior change [18,19]. Second, implementation outcomes were identified, which is important per understanding the “black box” of interventions [44] that will hopefully be able to add information to explain the effectiveness outcome findings.

Despite the strengths of this protocol, there are some limitations to acknowledge. The first limitation of our protocol is that women were recruited immediately after they gave birth. An antenatal recruitment process was also considered; however, due to the late arrival of many women in labor to the hospital wards, this was deemed not feasible by the nurse-midwives hired as research assistants for recruitment. In addition, earlier recruitment in the antenatal period (ie, before labor) was deemed not feasible as the messages are based on the date of the infant’s birth, thus would require the mother to inform this study when they had given birth. As a one-way intervention, this two-way contact was not feasible. This limitation may influence which women participate; however, the research assistants hired for recruitment were nurse midwives and thus were familiar with the clinical setting. They will be able to encourage participants to take breaks during the verbal interview and participants will be encouraged to continue baby care through the interview.

A second limitation of our study is that we are unable to collect information on participants who declined to participate. This will limit the ability to evaluate the recruitment process and representativity of our sample.

A third limitation of our study is that TextIt and Twilio do not report on the “read” or “unread” status of the messages. While it does report on the number of messages sent, delivered, or undelivered (error), whether the messages were read by participants and when is unknown. This may influence the implementation evaluation as the results will be unable to be controlled for this implementation variable. However, as part of the implementation evaluation, women are asked to recall how many messages they remember receiving per day and over how many days. Feedback on their perception of the messages is also requested, which will provide insight into what mothers recall.

A fourth limitation is that ECEM-TZ is not monitored in real time, and it is only a one-way intervention and thus, cannot be used as a clinical care tool. It will be made clear to participants that ECEM-TZ is not able to respond to clinical care questions as well as an automatic message is programmed to be sent to the mothers telling them to seek care if participants engage with the text messages. Nevertheless, despite these limitations, ECEM-TZ has a strong potential to improve outcomes for mothers in Tanzania while reducing the burden on health care providers.

### Future Directions

To expand this work, it will be important to explore potential challenges related to rural versus urban locale. This study recruited from Dar es Salaam, the commercial capital of Tanzania. For broad-scale expansion, exploring potential challenges in nonurban areas will be important. In considering postnatal care use in Tanzania more broadly, several studies have attributed higher use of postnatal care to higher maternal education [45,46], living in an urban area [45-47], receiving counseling from a health care provider to attend [45-47], higher socioeconomic status [46,47], and having trust in the health system [45]. Thus, expanding ECEM-TZ to other areas of Tanzania, including rural and remote areas, will need further research to understand how best to implement the program.

Future work should also consider including other caregivers such as fathers and grandparents in receiving such an mHealth intervention. Other caregivers have been found to play an important role in seeking postnatal care due to cultural norms [48]. Additionally, fathers in Tanzania have been found to struggle with psychosocial and mental health concerns during the perinatal and early childhood period [49]. Thus, an mHealth intervention that targets mothers as well as other caregivers is a warranted future direction.

## Abbreviations

CONSORT: Consolidated Standards of Reporting Trials
ECEM-TZ: Essential Coaching for Every Mother—Tanzania
EPDS: Edinburgh Postnatal Depression Scale
GAD: Generalized Anxiety Disorder
ITT: intention-to-treat analysis
LMIC: low- and middle-income country
mHealth: mobile health
RCT: randomized controlled trial
REDCap: Research Electronic Data Capture

## Data Availability

Data sharing does not apply to this paper, as the trial is ongoing.
